# The continuous adverse impact of COVID-19 on temporomandibular disorders and bruxism: comparison of pre- during- and post-pandemic time periods

**DOI:** 10.1186/s12903-023-03447-4

**Published:** 2023-10-04

**Authors:** Tamar Shalev-Antsel, Orit Winocur-Arias, Pessia Friedman-Rubin, Guy Naim, Lihi Keren, Ilana Eli, Alona Emodi-Perlman

**Affiliations:** 1 Department of Oral Rehabilitation, The Maurice and Gabriela Goldschleger School of Dental Medicine, Faculty of Medicine, Tel Aviv University, Tel Aviv, 6139001 Israel; 2 Department of Oral Pathology and Oral Medicine, The Maurice and Gabriela Goldschleger School of Dental Medicine, Faculty of Medicine, Tel Aviv University, Tel Aviv, 6139001 Israel; 3 The Maurice and Gabriela Goldschleger School of Dental Medicine, Faculty of Medicine, Tel Aviv University, Tel Aviv, 6139001 Israel

**Keywords:** COVID-19 pandemic, Temporomandibular Disorders, Bruxism, Gender

## Abstract

**Introduction:**

Some of the conditions affected by the COVID-19 pandemic were Temporomandibular Disorders (TMD) and bruxism. The present study compares the effect of the pandemic on TMD and bruxism (sleep and awake) in three time periods: before the pandemic (pre-COV), during the pandemic (during-COV) and after the pandemic subsided (post-COVR).

**Material and Methods:**

A total of 587 adult patients (108 in the pre-COV group, 180 in the during-COV group and 252 in the post-COVR group) who arrived for a routine dental treatment between October 2018 and January 2023 were evaluated according to Axis I diagnosis of the Diagnostic Criteria for Temporomandibular Disorders (DC/TMD). Each patient received a DC/TMD Axis I diagnosis as follows: (i) Painful TMD (defined by the presence of at least one of the following - local myalgia, myofascial pain with referral, arthralgia or headache attributed to TMD); (ii) Non painful TMD (defined by the presence of disc displacement with/without reduction, degenerative joint disorders and/or dislocation), (iii) Possible sleep bruxism (SB) and/or (iv) Possible awake bruxism (AB).

**Statistical methods:**

Logistic regression analyses were conducted to establish the impact of time and gender on the prospects of painful TMD, non-painful TMD, SB and AB.

**Results:**

The odds of subjects to be diagnosed with painful TMD at the post-COVR era were 3.3 times higher compared to the pre-pandemic time period (pre-COV, 95% C.I. 1.438–7.585). The odds of subjects to be diagnosed with non-painful TMD during-COV were 4 times higher compared to the pre-COV era (95% C.I. 1.332–12.542). The odds of subjects to present possible SB at post-COVR were 2.7 times higher compared to pre-pandemic (pre-COV, 95% C.I. 1.258–5.889, p < 0.05) and the odds to present possible AB after the pandemic subsided (post-COVR) were 3.2 times compared to the pre-pandemic period (95% C.I. 1.496–6.949). The odds of female subjects to be diagnosed with either painful or non-painful TMD were 3.7–4.4 times higher, compared to males.

**Conclusions:**

Results indicate that with regard to TMD and bruxism the pandemic adverse effects persist also after COVID-19 subsides and the restrictions caused by it are abolished. Apparently, during the pandemic females were affected more seriously by painful and non-painful TMD than males.

## Introduction

Oral diseases and conditions have significant socio-economic impact in terms of healthcare costs, the absence from school or workand individuals’ daily lives and self-esteem [1]. One of the most common oral condition is Temporomandibular Disorders (TMD) which relate to a group of conditions causing pain and dysfunction in the masticatory muscles, the temporomandibular joint (TMJ) and their associated structures [2]. TMD is fairly common, with an overall prevalence of approximately 31% for adults/elderly and 11% for children/adolescents [3]. Thus, TMD may play a major role in the public health burden worldwide.

The COVID-19 pandemic dramatically altered the routine lives of communities around the world inducing stress, anxiety, depression and sleep problems and causing severe social, mental and emotional health threats [4, 5]. Among oral syndromes affected by the situation were TMD and bruxism (sleep and awake) [2, 6, 7].

The most common features of TMD are regional pain, limited jaw movements and acoustic sounds from the TMJ during motion [8]. TMD is usually diagnosed through Diagnostic Criteria for Temporomandibular Disorders (DC/TMD) which are based on the criteria of the international DC/TMD consortium (http://rdc-tmdinternational.or). The etiology of TMD is multifactorial and includes biomechenichal, genetic, hormonal and psychosocial risk factors [9]. A recent literature meta-analysis showed a correlation between posture and TMD [10]. Additional factors such as anxiety, stress, depression, coping strategies and catastrophizing influence the onset of TMD pain, as well as precipitate or prolong it [9].

TMD carry numerous adverse effects on subjects’ lives and require a comprehensive approach. Studies demonstrated significant effect of TMDs on subjects’ oral health-related quality of life [11]. Due to its complex nature treatment of TMD can be challenging. Conservative treatment approaches have been proposed such as physical therapy [12], low-level laser therapy and laser acupuncture therapy [13]. Additional first-line treatments include pharmacological drugs, occlusal splints, extracorporeal shockwave therapy, transcutaneous electrical nerve stimulation (TENS), and oxygen–ozone therapy [14].

Bruxism is defined as a repetitive jaw muscle activity characterized by clenching or grinding of the teeth and/or bracing or thrusting of the mandible [15]. In otherwise healthy individuals, bruxism should not be considered as a disorder but rather a behavior that may be a risk or a protective factor, for certain clinical consequences [15]. Bruxism is further separated into two distinct muscle behaviors according to whether they appear during wakefulness (Awake bruxism - AB) or during sleep (Sleep bruxism -SB) [15]. Bruxism diagnosis is graded as “possible” when it is based solely on self-report or diaries recalling the performance of such a behaviour. When evaluation includes clinical examination (intra and extra oral clinical signs such as masticatory muscle hypertrophy, linea alba or scalloped tongue) bruxism is graded as “probable”. A “definite” bruxism diagnosis is based on a polysomnographic recording with a simultaneous audio-video recording [15]. Both SB and AB may be affected by stress and anxiety [16–18] although some research shows that self-reported perceived stress and depression are not necessarily correlated with the intensity of SB [17].

The relationship between TMD and bruxism is complex and controversial [19–21]. Nevertheless, if muscle activity exceeds physiologic tolerance, breakdown in the stomatognathic system may occur and induce orofacial pain and TMD [22–24].

As in many other countries, the initial reaction of the State of Israel to the COVID − 19 pandemic was to impose several lockdown periods [25]. The country scored 86.28 on the Containment and Health Index developed by the Oxford Coronavirus Government Response Tracker project [26]. The index is based on school and workplace shutdowns, cancellation of public events, restrictions of public gatherings, shutdown of public transportation, stay-at-home requirements, restrictions on internal movements, international travel controls, testing policy, extent of contact tracing, requirements to wear face coverings, etc. [6]. The high index achieved by the State of Israel (one of the highest in the world) indicates severe restrictions on subjects’ everyday lives for a significant period of time.

One of the noticeable results of the COVID-19 pandemic was an increase in the report of TMD symptoms and bruxism, due to the stressogenic environment imposed by the pandemic and lockdowns [2, 6, 27, 28]. A previous study conducted on the general population in Israel showed that the COVID-19 pandemic caused significant adverse effects on the psycho-emotional status of the Israeli population, resulting in the intensification of their bruxism and TMD symptoms [6]. A recent systematic review, published in 2023, showed an agreement regarding the association between COVID-19 and increased incidence of TMD [28].

When the pandemic subsided the COVID-19 restrictions were gradually removed, allowing a complete return to the former routine of work, studies and social lives. But have TMD and bruxing subsided in full? The aim of the present study is to compare the effect of the pandemic on the prevalence of painful and non-painful TMD and of bruxism (AB, SB) in three time periods: before the pandemic, during the lockdown periods and after the pandemic subsided and social restrictions were abolished.

## Materials and methods

The study was planned as a cross sectional study and conducted according to a previously described protocol. [7]

### Population

Subjects who arrived for a routine dental treatment at the School of Dental Medicine, Tel Aviv University, between October 2018 and January 2023 were evaluated according to Axis I of the DC/TMD [29]. Neither of the subjects arrived for treatment with a specific complaint referring to TMD.

Exclusion criteria included subjects under the age of 18 years or over 65 and/or subjects suffering from neurological disturbances, a history of facial or cervical injury uncontrolled hormonal disease, neoplasm, psychiatric problems, patients under medication (e.g. myo-relaxants, anti-depressants, drug users).

Three study groups were defined according to time of admission and evaluation:

1. Subjects evaluated between October 2018 and February 2020 – the pre-pandemic group (**pre-COV**, No = 108).
2. Subjects evaluated between March 2020 and June 2021 during the social distancing period – the pandemic group (**during-COV**, No = 180).
3. Subjects evaluated between July 2021 and January 2023 after all social restrictions were abolished - the post COVID restrictions’ group (**post-COVR**, No = 251).

### Instruments

#### TMD evaluation

All subjects were clinically evaluated by senior dental students according to Axis I of the DC/TMD. The accuracy of the examination was verified by a senior faculty member who was officially calibrated and certified by the DC/TMD Training and Calibration Course at the Department of Orofacial Pain and Jaw function at the Faculty of Odontology, Malmö University, Sweden. Diagnosis of TMD was based on the criteria of the international DC/TMD consortium (http://rdc-tmdinternational.org). In order to obtain a final TMD diagnosis all subjects were also requested to complete the official Hebrew version of the Symptoms Questionnaire [29].

TMD was further defined as painful or non-painful:

Painful TMD (pain TMD) was defined when one of the following diagnoses was reached: local myalgia, myofascial pain with referral, arthralgia or headache attributed to TMD.

Non painful TMD (non-pain TMD) was defined when one of the following diagnoses was reached: disc displacement with/without reduction, degenerative joint disorders and dislocation.

#### Sleep and awake bruxism

SB and/or AB definition depended upon subjects’ self-report referring to bruxing behavior, as reported through the official Hebrew version of the Oral Behavior Checklist [29] (possible SB, possible AB).

Possible sleep bruxism (SB) was defined when subjects reported performing the behavior at a frequency of minimum 1–3 nights a week. [29]

Possible awake bruxism (AB) was defined when subjects reported at least one AB behavior listed in the Oral Behavior Checklist (i.e., grinding, clenching, pressing, touching or holding teeth, tightening muscles without clenching) [29].

The study was approved by the Tel Aviv University Institutional Ethical Committee prior to data collection (ID: 0005308-1).

### Statistical methods

Data were analysed using IBM SPSS statistics version 25.0. (SPSS, Inc., Chicago, IL USA). The level of statistical significance was set on 0.05.

Categorical variables were described as frequencies with percentages and appraised using Chi-square tests. Logistic regression analyses were conducted to establish the impact of time and gender on the prospects of pain TMD, non-pain TMD, SB and AB, as well as potential interaction effects between time and gender. Findings were reported as odds ratios with 95% confidence intervals.

## Results

### Descriptive data

A total of 587 adult subjects were included in the study. Details regarding the study groups are presented in Table 1. There were no differences among groups age- and/or gender wise.

**Table 1:** Demographic details of the study population

	Total (No = 539)	Pre-COV (No = 108)	During-COV (No = 180)	Post-COVR (No = 251)
Gender: Male Female	52.1% 47.9%	54.6% 45.4%	53.3% 46.67%	50.2% 49.8%
Age (years): Mean (± SD)	34.5 (± 12.5)	35 (± 11.45)	36.0 (± 12.92)	33.2 (± 12.45)

In general females were diagnosed more often with both painful and non-painful TMD, as well as presented more possible SB and/or AB behavior, as compared to males (Table 2).

**Table 2:** Distribution of positive cases according to gender

Gender*/Variable	Female	Male	p**
Pain TMD	44.7%	24.9%	0.000
Non-pain TMD	24.4%	17.1%	0.04
SB	38.0%	28.9%	0.02
AB	49.4%	33.2%	0.000

*percent of positive cases within gender

**Fisher exact test (2 sided)

The prevalence of painful TMD, non-painful TMD, SB and AB are presented in Table 3.

**Table 3:** Prevalence of TMD and bruxism according to time period

Time*/Variable	pre-COV	during-COV	post-COVR
Pain TMD	24.1%	21.1%	48.4%
Non-pain TMD	14.8%	21.1%	22.8%
SB	14.8%	31.6%	42.4%
AB	15.7%	44.6%	49.2%

*percent of positive cases (in the entire population, males and females) within time points

### Logistic regression analyses

#### The impact of time and gender on the prospects of painful TMD (Fig. 1)

Main effects of time and gender could be observed. The prospects of subjects to be diagnosed with painful TMD in the post-COVR era was 3.3 times higher than the pre-COV era (odds ratio = 3.303, 95% C.I. 1.438–7.585). There were no differences in the prospects of painful TMD between the periods of during-COV and pre-COV.

The odds of female subjects to be diagnosed with painful TMD were 3.7 times compared to males (odds ratio = 3.702, 95% C.I. 1.439–9.523).

No interactions between time and gender were observed.

**Fig. 1:**
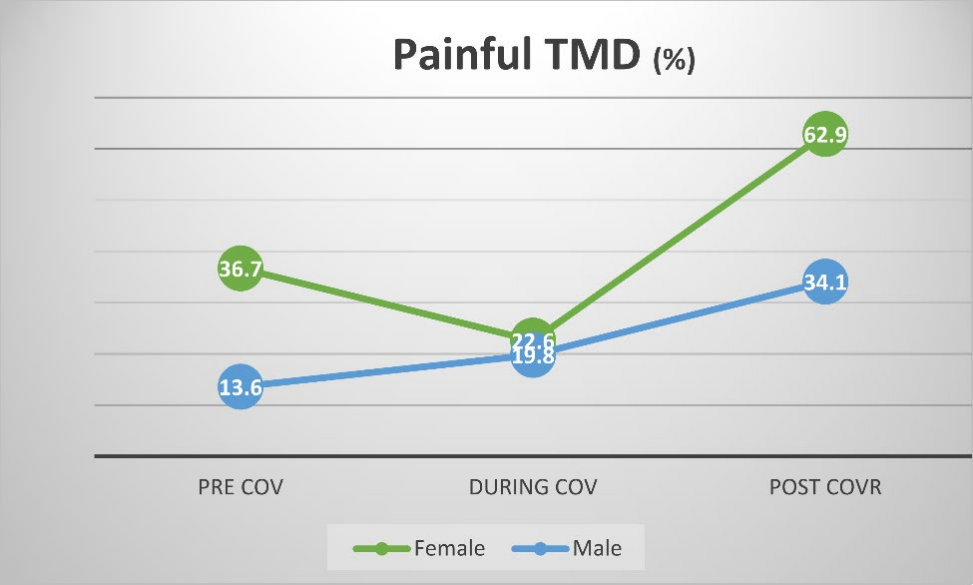
Prevalence of painful TMD according to gender and time period* *percent of positive cases within group.

#### The impact of time and gender on the prospects of non-painful TMD (Fig. 2)

Main effects of time and gender were observed. The prospects of subjects to be diagnosed with non-painful TMD during-COV were 4 times higher than in the pre-COV era (odds ratio = 4.088, 95% C.I. 1.332–12.542). There were no differences in the prospects of subjects to be diagnosed with non-painful TMD between the post-COVR and pre-COV time periods.

The prospects of females to be diagnosed with non-painful TMD were 4.4 times higher than in males (odds ratio = 4.459, 95% C.I. 1.335–14.893).

A significant interaction between time and gender could be observed, with the odds of female subjects to be diagnosed with non-painful TMD were actually lower at the during-COV period than at the pre-COV period (odds ratio = 0.177, 95% C.I. 0.043–0.724).

**Fig. 2:**
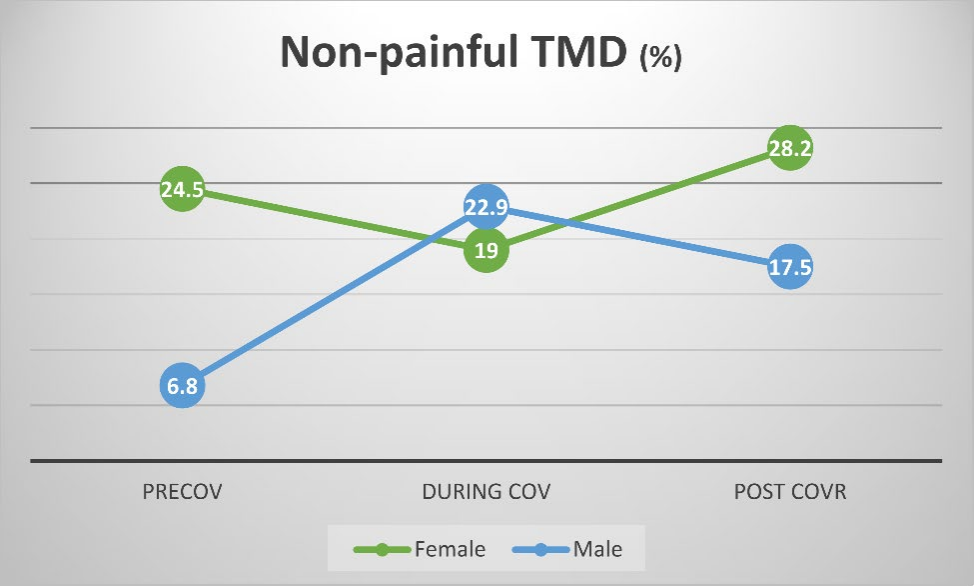
Prevalence of non-painful TMD according to gender and time period* *percent of positive cases within group.

#### The impact of time and gender on the prospects of SB (Fig. 3)

A main effect of time could be observed. The odds of subjects to present possible SB at post-COVR were 2.7 times higher than pre-COV (odds ratio = 2.722, 95% C.I. 1.258–5.889, p < 0.05). There was no difference between the during-COV and the pre-COV time periods.

Neither a main effect of gender nor interactions between time and gender could be detected.

**Fig. 3:**
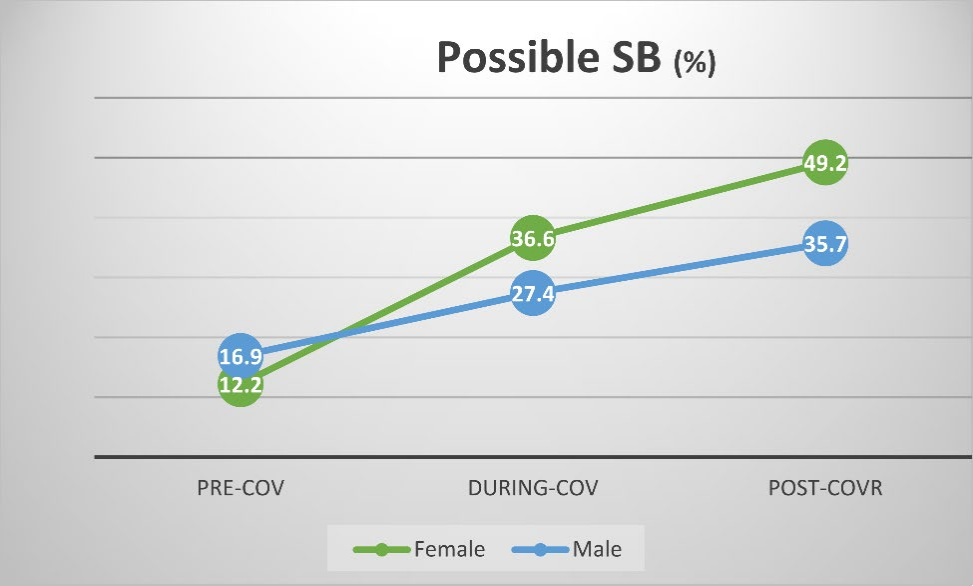
Prevalence of possible SB according to gender and time period* *percent of positive cases within group.

#### The impact of time and gender on the prospects of AB (Fig. 4)

A main effect of time was observed. The odds of subjects to be diagnosed with possible AB during the pandemic were 2.6 times higher than before the pandemic (odds ratio = 2.609, 95% C.I. 1.171–5.808). The prospects of subjects to be diagnosed with AB after COVID restrictions were abolished (post-COVR) were 3.2 higher than before the pandemic (odds ratio = 3.24, 95% C.I. 1.496–6.949).

Neither a main effect of gender nor interactions between time and gender could be detected.

**Fig. 4:**
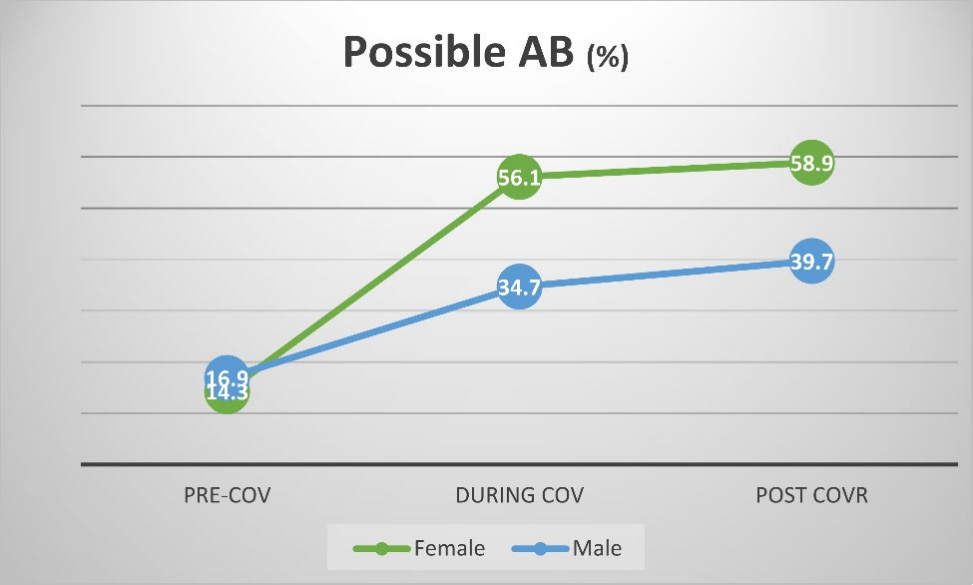
Prevalence of possible AB according to gender and time period* *percent of positive cases within group.

## Discussion

Besides posing severe infectious hazards, the COVID-19 pandemic presented numerous additional adverse effects on the population’s health and wellbeing. At its outbreak the pandemic involved uncertainty concerning the modes of virus spread and treatment, insufficient availability of local health services and lack of vaccine or efficient drugs for treatment [30]. The most popular policy to fight the new threat was imposing social distancing and partial to total lockdowns. The situation burdened people not only with immediate severe health threats but also with economic uncertainty and social isolation, causing further potential deleterious effects on their mental and physical health [31].

It is now well supported that the pandemic resulted in increased levels of stress, depression and anxiety all around the globe [32–35]. Santomauro et al. reported an increased prevalence of depression and anxiety in both sexes, across all ages, in 204 countries [36]. These emotional and psychosocial factors exhibited a potential risk factor for the development and rise of TMD [37–40]. Indeed, an intensification of bruxism and TMD symptoms was observed during the pandemic [6, 7, 41]. The assumption was that once the pandemic subsides and life returns to its pre-pandemic routine, bruxing and TMD symptoms subside as well and return to their pre-pandemic level. Regretfully, the present results do not support this assumption.

Stress is one of the psychological factors involved in TMD pathophysiology. Increased levels of stress in patients with TMD are associated with elevated levels of cortisol, hyperactivity of the Hypothalamus-Pituitary-Adrenal axis and increased bio-electric activity of the masticatory muscles [42]. Additional origins of TMD pain may be due to voluntary muscle contraction leading to syndromes such as chronic fatigue syndrome (CFS) and fibromyalgia which are frequently associated with TMD [43]. A recent comprehensive review showed that central sensitization and the inhibitory system of descending pain play a role in the TMD clinical pattern [14]. Central sensitization can lead to the development of increased pain sensation from noxious or non-noxious stimuli (allodynia) [44] which may possibly persist after the initial trigger comes to its end.

Present results indicate not only a lack of decrease in TMD, SB and/or AB, but an actual increase in the percentage of subjects presenting TMD and SB/AB after all restrictions caused by the pandemic were finally abolished. The raise in TMD may be a result of increase in oral behaviors during the pandemic, as shown in a previous publication [7]. An ongoing overload of masticatory muscle activity may have exceeded the individual’s physiological tolerance and aggravated or triggered stomatognathic conditions such as painful and non-painful TMD. The influence of emotional status on TMD may also have been caused also by an upregulated hypothalamic-pituitary-adrenal axis in TMD patients (compared to non TMD patients) as suggested by some authors [39, 45].

The odds of possible SB and AB were almost three-fold higher in the post-pandemic era than before the pandemic. This is in agreement with Osses-Anguita et al. [41] who concluded that the COVID-19 pandemic affected sleep and awake bruxers. The authors explained some of the differences apparent in their results with differences in subjects’ personality traits and coping styles. According to Soto et al. [46] awake oral behaviors may play a protective role in an ongoing stressful situation, such as the one caused by the pandemic. Thus, the increase in awake masticatory muscle activity during the pandemic may have served as an adaptive stress coping tool to reduce stress by releasing cortisol and producing salivary ingredient which reduces negative mood [46–48].

Polysomnographic studies show that SB is associated with transient arousal response, poor sleep quality and altered sleep architecture [49]. A survey conducted during the early stages of the pandemic showed that over half of the participants reported an increase in difficulty falling asleep and disturbed and/or restless sleep [2]. A recent meta-analysis showed that sleep disturbances were higher during lockdown compared to no lockdown and that four in every ten individuals reported a sleep problem during the pandemic [50]. This could have served as an additional trigger for the increase in painful TMD, AB and SB during the pandemic.

The finding that females were affected by the pandemic more profoundly than males is well documented [51]. Women exhibit higher stress sensitivity and different stress response than men [52]. Indeed, during the pandemic women reported higher prevalence of general anxiety and reduced sleep quality [53]. Present results indicate that females’ prospects to be diagnosed with painful or non-painful TMD were substantially higher than males’ (odds ratio 3.7 and 4.4, respectively). The results for non-painful TMD are somewhat more complex showing an interaction between time and gender (females actually experienced less non-painful TMD during the pandemic than before the pandemic). This may be due to the difference in the profile of patients arriving to dental clinics during the pandemic. During lockdown periods dental clinics provided mainly urgent treatment for acute painful conditions and most subjects avoided in-office consultations for non-painful conditions [54]. The fact that the prevalence of painful and non-painful TMD during the pandemic was almost similar in both genders further supports this notion. Additional explanation may origin in the modulating effect of psychosocial factors on the development and/or aggravation of painful TMD, contrary to non-painful TMD that results from other etiological risk factors [55].

Yap et al. suggested that the prevalence of pain-related and intra-articular (non-painful) TMD among East Asian patients was not significantly affected by the COVID-19 pandemic and suggested that sex and age play a more crucial role in the development of painful and non-painful TMD than the pandemic [56]. It is noteworthy that Yap et al. refer to two time periods only (before and during the pandemic). The present study evaluated subjects in three time periods: pre-, during- and post-pandemic, showing that although life has seemingly returned to its pre-pandemic routine, the pandemic’s adverse effects continue and even increase in magnitude.

The present study suggests that the effect of the pandemic on TMD, SB and AB may be longer and more profound than initially assumed. Although the end of COVID-19 as a global health emergency was officially declared on May 2023 [57] its mental and physical burden may still be l present. A post pandemic syndrome, probably caused by a long-term effect of ongoing rise in muscle activity, might induce long term effects on myofascial pain. Further studies should be carried out to evaluate the long-term effect of the COVID pandemic on orofacial pain and bruxing behavior.

### Limitations

As in other cross-sectional studies, the present study is unable to make a causal inference and is susceptible to a sampling bias. Applying a longitudinal, long-term design, would enable a more profound detection of trends and relationships within the data collected. Furthermore, DC/TMD Axis II was not included in the study, so that results could not refer to a possible TMD/bruxism psychosocial association. Finally, bruxism was evaluated only through self-report which allowed reaching a definition of possible SB or AB. Use of the recently introduced standardized tool for bruxism assessment (STAB) would have allowed a better screening of AB and SB and assessing risk factors and possible consequences of these masticatory behaviors [58].

## Conclusions

The adverse effects of the COVID-19 pandemic on TMD, SB and AB last longer and may be more profound than initially assumed. Apparently, females were more seriously affected by the pandemic than males.

## Data Availability

the datasets used and/or analyzed during the current study is available from the corresponding author on reasonable request.
